# Social capital dynamics and health in mid to later life: findings from Australia

**DOI:** 10.1007/s11136-017-1655-9

**Published:** 2017-07-26

**Authors:** Vasoontara Yiengprugsawan, Jennifer Welsh, Hal Kendig

**Affiliations:** 1 Centre for Research on Ageing, Health and Wellbeing, Research School of Population Health (CRAHW), The Australian National University, Canberra, Australia; 2 The Australian Research Council Centre of Excellence in Population Ageing Research (CEPAR), Canberra, Australia; 3 National Centre for Epidemiology and Population Health, Research School of Population Health (NCEPH), The Australian National University, Canberra, Australia

**Keywords:** Social capital, Social participation, Trust, Self-rated health, Ageing, Middle and older adults

## Abstract

**Purpose:**

The influence of social capital has been shown to improve health and wellbeing. This study investigates the relationship between changes in social capital and health outcomes during a 6-year follow-up in mid to later life in Australia.

**Methods:**

Nationally representative data from the Household, Income and Labour Dynamics in Australia (HILDA) survey included participants aged 45 years and over who responded in 2006, 2010 and 2012 (*N* = 3606). Each of the three components of social capital (connectedness, trust and participation) was measured in Waves 2006 and 2010 and categorised as: ‘never low’, ‘transitioned to low’, ‘transitioned out of low’ and ‘consistently low’. Health outcomes in 2012 included self-rated overall health, physical functioning, and mental health based on the Short Form 36-item health survey (SF-36). Multivariable logistic regression assessed changes in social capital (measured in 2006 and 2010) predicted poor health (measured in 2012), adjusting for covariates.

**Results:**

Consistently low trust was significantly associated with higher odds of transitions into poor physical functioning (AOR 1.54; 95% Confidence Interval 1.06–1.22), poor mental health (AOR 1.59; 95% CI 1.08–2.36) and poor self-rated health (AOR 1.86; 95% CI 1.27–2.72). Transition into low trust was also a predictor of poor self-rated health after adjusting for covariates (AOR 1.74; 95% CI 1.11–2.73). Changes in social connectedness in both directions (transitioned out of and into low) were statistically associated with poor self-rated health (AORs 1.40; 95% CI 1.00–1.97 and 1.61; 95% CI 1.11–2.34, respectively) after adjusting for confounders as well as other social capital components.

**Conclusions:**

Our longitudinal findings reveal social capital dynamics and effects on health in mid to later life. Social trust and connectedness could be important enablers for older persons to be more active in the community and potentially benefit their health and wellbeing over time.

**Electronic supplementary material:**

The online version of this article (doi:10.1007/s11136-017-1655-9) contains supplementary material, which is available to authorized users.

## Introduction

Putnam’s seminal work on social capital, building from concepts of social democracy [1], has been applied in a range of empirical work linking social bonding to beneficial health outcomes [2] and overall life satisfaction [3]. Much of this work has focused on psycho-social resources—notably trust, social support, social networks and reciprocity while community dimensions have also been addressed primarily in terms of social inequalities and spatial segregation [4, 5].

In the past few decades, emerging research in Western countries has focused on social capital and its role in later life [6–9]. A cross-national study in Europe has reported that, regardless of the levels of social trust and social networks, there were similar associations between social capital and self-assessed health among older adults in Finland, Poland and Spain [6]. Another comparative study among the elderly reported that low trust was associated with adverse self-rated health in both the US and Germany; in addition, lack of social participation was also associated with poor self-rated health and depression in Germany [7]. International reviews of public policy have argued for improving social capital as an important strategy for reducing social exclusion and inequality among disadvantaged older people [8].

In Australia, there have been calls to consider social capital as part of the public health agenda [10] including monitoring population health [11]. A cross-sectional study in two suburbs of Adelaide found that those who were better off materially had better access to social capital; further, perceived material advantage as well as social capital was associated with mental and physical health [12]. An early national study found that measures of social capital and perceived material wellbeing predicted mental, but not physical health [13]. Another national cross-sectional Australian study has shown that structural (community participation) and cognitive (social cohesion) components of social capital related to general health, mental health and physical functioning [13]. However, empirical longitudinal data are limited especially for older populations.

The aim of this research is to provide longitudinal evidence on the changes in social capital and effects on health outcomes in mid to later life. In particular, we set out to investigate the relationship between three components of social capital (connectedness, trust, participation) and effects on vulnerability in terms of health during a 6-year follow-up among participants aged 45 years and over in Australia.

## Methods

### Data and sample

This study used nationally representative data from the Household, Income and Labour Dynamics in Australia (HILDA) survey. HILDA data are primarily collected using face to face or telephone interviews but information on more sensitive topics, including social attitudes and health is collected using a mail back self-completed questionnaire. This study is based on Waves 6, 10 and 12 (collected in 2006, 2010 and 2012) because of special topic modules relating to social capital in these waves. Respondents were included in this study if they were aged 45 years or older in Wave 6 (2006) and returned their questionnaire in all the three waves (*N* = 3606). Appendix 1 includes information on sample and inclusion criteria in the supplementary data.

### Measures

#### Exposure-social capital

We measured three components of social capital: “low connectedness”—infrequent contact with friends or relatives or perceptions that neighbours are unwilling to help; “low trust”—low generalised trust; and “low participation”’—no club membership and only infrequent attendance at community events (more information in Appendix 2 in the Supplementary Material). Each was measured in Waves 6 and 10, allowing us to further categorise components according to transitions between waves: ‘never low’, ‘transitioned to low’, ‘transitioned out of low’ and ‘consistently low’.

#### Health outcomes

We focused on three measures: self-rated overall health, physical functioning and mental health in Wave 12 based on the international standardised medical outcomes study Short Form 36-item health survey [14]. Respondents were considered to have poor self-rated health if they reported their overall health as ‘poor’ or ‘fair’ or poor physical functioning or mental health if their score was in the bottom 20% of scores for their age group (Appendix 2 in the Supplementary Material).

#### Covariates

In order to assess the main effects of social capital, the following potential confounding variables from Wave 6 were grouped into categories: sex, age groups, marital status, employment status, household equivalised annual income, region of residence, number of people in the household and whether the respondent had a long-term health condition.

### Statistical approach

Multivariable logistic regression assessed the extent to which transitions in connectedness, trust and participation (measured in Waves 6 and 10) predicted poor health (measured in Wave 12) and taking into account covariates including baseline health from Wave 6. Analyses were run separately for each health outcome. Respondents reporting poor health (assessed with the cut points noted above) at the baseline of the study in 2006 were excluded from the analysis. Models were first adjusted for confounders (Model 1) and then additionally for other components of social capital (Model 2). Data were weighted to the population [15].

## Results

Characteristics of the sample are presented in Table 1: approximately 75% aged between 45 and 65 years, 17% were 65–74 years, and 8% were 75+ years. Across the three components of social capital: low social connectedness 34%; low trust 29%, and low participation 23% were reported in 2006. The number and weighted percent of respondents in each of social capital dynamics for social connectedness, trust, and participation between 2006 and 2012 and the multivariable associations with health outcomes are shown in Table 2.

**Table 1:** Study sample, household income labour dynamics in Australia survey, 2006

	Total (%)	Categories of low social capital (column %)		
		Low connection	Low trust	Low participation
Attributes				
Age groups				
45–54	1489 (41)	525 (45)	502 (50)	344 (43)
55–64	1156 (33)	361 (34)	261 (30)	251 (34)
65–74	681 (17)	184 (14)	177 (16)	141 (15)
75+	280 (8)	64 (7)	50 (4)	51 (7)
Sex				
Male	1696 (49)	548 (48)	476 (49)	403 (52)
Female	1910 (51)	586 (52)	514 (51)	384 (48)
Marital status				
Married/de facto	2652 (77)	800 (73)	690 (72)	552 (72)
Single	954 (23)	334 (27)	300 (28)	235 (28)
Employment status				
Full-time	1378 (38)	495 (44)	392 (40)	332 (44)
Part-time	654 (17)	200 (15)	164 (15)	106 (11)
Unemployed	50 (1)	21 (1)	26 (2)	15 (1)
Not in workforce	1524 (44)	418 (40)	408 (44)	334 (43)
Health condition				
Yes	1247 (36)	416 (40)	395 (42)	321 (41)
No	2359 (65)	718 (60)	595 (58)	466 (59)
Residence				
Major urban	2042 (61)	687 (66)	568 (63)	471 (65)
Other urban	862 (22)	257 (20)	255 (23)	186 (21)
Rural	702 (17)	190 (14)	167 (14)	130 (14)
Income (quintiles)				
1 poorest	838 (21)	263 (21)	263 (25)	207 (25)
2	654 (18)	212 (20)	191 (21)	148 (19)
3	639 (18)	210 (19)	182 (18)	144 (20)
4	714 (21)	205 (19)	190 (21)	132 (15)
5 richest	761 (22)	244 (21)	164 (16)	156 (21)
Number of people				
1	685 (13)	213 (12)	191 (12)	161 (13)
2	1752 (47)	512 (42)	461 (45)	381 (47)
3	488 (18)	154 (21)	136 (19)	112 (22)
4+	681 (21)	255 (25)	202 (23)	133 (18)
Social capital				
Connectedness				
High–moderate	2472 (66)	–	565 (52)	394 (48)
Low	1134 (34)	–	425 (48)	393 (52)
Trust				
High–moderate	2616 (71)	709 (60)	–	471 (59)
Low	990 (29)	425 (40)	–	316 (41)
Participation				
High–moderate	2819 (77)	741 (65)	674 (67)	–
Low	787 (23)	393 (35)	316 (33)	–
Health outcomes				
Physical functioning				
High–moderate	2879 (78)	852 (70)	714 (68)	560 (69)
Poor	727 (22)	282 (30)	276 (32)	227 (31)
Poor mental health				
High–moderate	2792 (76)	785 (66)	649 (62)	525 (64)
Poor	814 (24)	349 (34)	341 (38)	262 (36)
Self-rated health				
Excellent–good	2872 (78)	842 (69.8)	704 (64)	515 (64)
Poor–fair	734 (22)	292 (30.2)	286 (36)	272 (36)

Numbers are based on 3606 respondents who met the inclusion criteria

**Table 2:** Multivariable associations predicting change into poor health, excluding those with poor health at baseline and controlling for baseline health, household income labour dynamics in Australia survey

Social capital dynamics	*N* (%)	Adjusted odds ratios [95% Confidence Interval] by each adverse health outcome, 2012					
2006 and 2010 categories		Poor physical functioning (*n* = 2879)		Poor mental health (*n* = 2792)		Poor self-rated health (*n* = 2872)	
		Model 1	Model 2	Model 1	Model 2	Model 1	Model 2
Connectedness							
Never low	2021 (54)	1.00	1.00	1.00	1.00	1.00	1.00
Transitioned out of low	538 (16)	1.09 [0.77–1.54]	1.04 [0.73–1.47]	1.23 [0.72–2.10]	1.10 [0.61–1.97]	**1.53 [1.10–2.14]**	**1.40 [1.00–1.97]**
Transitioned into low	451 (12)	1.18 [0.77–1.80]	1.11 [0.71–1.72]	**1.54 [1.02–2.33]**	1.38 [0.90–2.10]	**1.76 [1.24–2.52]**	**1.61 [1.11–2.34]**
Consistently low	59 (18)	1.36 [0.90–2.07]	1.22 [0.77–1.93]	1.16 [0.78–1.72]	0.96 [0.63–1.46]	1.05 [0.71–1.56]	0.87 [0.59–1.29]
Trust							
Never low	2275 (61)	1.00	1.00	1.00	1.00	1.00	1.00
Transitioned out of low	483 (14)	0.94 [0.64–1.38]	0.90 [0.61–1.31]	1.41 [0.88–2.26]	1.38 [0.86–2.21]	1.07 [0.71–1.59]	1.03 [0.69–1.55]
Transitioned into low	341 (10)	1.53 [0.96–2.44]	1.47 [0.93–2.33]	1.36 [0.83–2.23]	1.31 [0.78–2.18]	**1.79 [1.14–2.80]**	**1.74 [1.11–2.73]**
Consistently low	507 (15)	**1.64 [1.15–2.32]**	**1.54 [1.06–2.22]**	**1.69 [1.14–2.49]**	**1.59 [1.08–2.36]**	**1.96 [1.35–2.85]**	**1.86 [1.27–2.72]**
Participation							
Never low	2529 (68)	1.00	1.00	1.00	1.00	1.00	1.00
Transitioned out of low	367 (10)	1.14 [0.73–1.77]	1.06 [0.67–1.68]	1.37 [0.89–2.10]	1.30 [0.83–2.04]	1.26 [0.85–1.88]	1.14 [0.75–1.72]
Transitioned into low	290 (9)	1.22 [0.75–1.99]	1.14 [0.69–1.89]	1.35 [0.84–2.15]	1.22 [0.77–1.93]	1.33 [0.87–2.03]	1.17 [0.76–1.79]
Consistently low	420 (13)	1.35 [0.94–1.93]	1.26 [0.85–1.84]	1.48 [0.96–2.29]	1.41 [0.89–2.23]	**1.53 [1.02–2.31]**	1.38 [0.89–2.14]

Bold values indicate statistically significance results (*p* < 0.05)

Respondents reporting poor health (assessed with the cut points) at the baseline of the study in 2006 were excluded from the analysis. Estimates were weighted to the population and were adjusted for the survey design. Model 1 is adjusted for: age groups, sex, marital status, employment status, health condition, residence, number of people in the household, and income quintiles. Model 2 is further adjusted for all components of social capital simultaneously

Transition into low connectedness between 2006 and 2010 was associated with poor mental health (Adjusted Odds Ratio, AOR 1.54; 95% Confidence Interval 1.02–2.33). However, once adjusted for trust and participation dynamics, the effect size was still high but no longer statistically significant (AOR 1.38; 95% CI 0.90–2.10). Transitions out of and into low connectedness were significant predictors in reporting poor self-rated health (AORs 1.53; 95% CI 1.10–2.14 and 1.76; 95% CI 1.24–2.52, respectively) after adjusting for confounders as well as other social capital components (AORs 1.40; 95% CI 1.00–1.97 and 1.61; 95% CI 1.11–2.34, respectively).

Low trust was robustly associated with all three health outcomes with an observed gradient of adverse health outcomes from never low, transitioned into low, and consistently low. In particular, consistently low trust were significantly associated with higher odds of transitions into poor physical functioning (AOR 1.54; 95% CI 1.06–1.22), poor mental health (AOR 1.59; 95% CI 1.08–2.36) and poor self-rated health (AOR 1.86; 95% CI 1.27–2.72). Transition into low trust was also a predictor of poor self-rated health after adjusting for covariates (AOR 1.74; 95% CI 1.11–2.73). Consistently, low social participation was statistically associated with poor self-rated health (AOR 1.53; 95% CI 1.02–2.31). However, after further adjusting for trust and connectedness dynamics, the associations attenuated and were no longer statistically significant.

## Discussion

We report findings on social capital dynamics and health among nationally representative samples aged 45 years and older in Australia. Across the three social capital components, consistently low social trust dynamics were the strongest predictors for all outcomes especially for poor self-rated health. Notably, changes in social connectedness in both directions (transitioned into and out of low) were statistically associated with poor self-rated health. This strong effect could reflect the relationship between social connection and self-perceived health. Besides social trust, other transitions were not statistically significant adjusting for other social capital components.

Our findings on social trust predicting self-rated health were in line with a longitudinal study in a sample of three ageing cohorts in Finland which reported that stability and change of high levels of trust over three years have important effects on self-rated health [16]. However, a comparative study has shown that Finland generally had almost twice the higher proportion of trust as compared to Spain and Poland [6]. Our older Australian samples reported similar proportions of trust levels and have also shown similar association with health outcomes to the latter two countries (e.g. significant relationship between trust and self-rated health in both Spain and Poland).

Our findings provide international evidence on the role of social capital in later life [9]. In particular, having trust could be an important enabler for older persons to be more active in the community. Social capital through participation could alleviate loneliness among older persons which in turn could help to improve their health and wellbeing. Promoting social capital and facilitating formal and informal social networks can be an effective health promotion strategy for older populations [12].

Some considerations for this study include firstly the strength of representative national samples with an array of sociodemographic and health covariates which could be taken into account in the analyses. Secondly, there might be bi-direction relationships between social capital and health [16, 17] and consequently to minimise reverse causality effects, our analyses were restricted to participants who did not have poor health at the baseline. Thirdly, we also investigated generalised trust, connectedness, and participation as our social capital measures; however, these measures may not capture all dimensions of social relationships. In future studies with larger samples and stronger cross-national comparative dimensions, such as the longitudinal Australian survey of ageing populations now under development, it would be possible to better understand the influence of varying personal and social context—for example gender, age, life history and social class variations at different points across later life [18].

In Australia, there has been increasing research and advocacy on behalf of ‘ageing well’, that is, the positive dimensions of health and wellbeing, noting the attitudinal and structural barriers facing people in mid to later life [19]. This action can include psycho-social interventions with vulnerable older people and extend to social actions such as addressing age discrimination in the workplace as well as related social policies [20]. New ways of conceptualising challenges and opportunities over the life course can greatly benefit Australia in the midst of rapid population ageing.

## Electronic supplementary material

Below is the link to the electronic supplementary material.

Supplementary material 1 (DOCX 14 kb)
