# Supplementary material for: Social capital dynamics and health in mid to later life: findings from Australia
Source: Qual Life Res. 2017 Jul 26;27(5):1277–82. doi: 10.1007/s11136-017-1655-9 (PMC5891551; doi:10.1007/s11136-017-1655-9)
Supplement: Supplementary file 1 — Supplementary material 1 (DOCX 14 kb) [file 11136_2017_1655_MOESM1_ESM.docx]

**Appendix 1 Sample and inclusion criteria**

The Household, Income and Labour Dynamics in Australia (HILDA) Survey is a longitudinal and nationally representative survey of Australian adults aged 15 years and over which collects a range of data relevant to life in Australia, including social attitudes and health. Respondents were included in this study if they were aged 45 years or older at the baseline of our study (wave 6) and returned their self-completion questionnaire in Waves 6, 10 and 12. Respondents were also excluded from analyses if they reported poor health at the baseline of the study or reported missing data on any of the exposure variables. Figure S1 describes the participation flow chart.


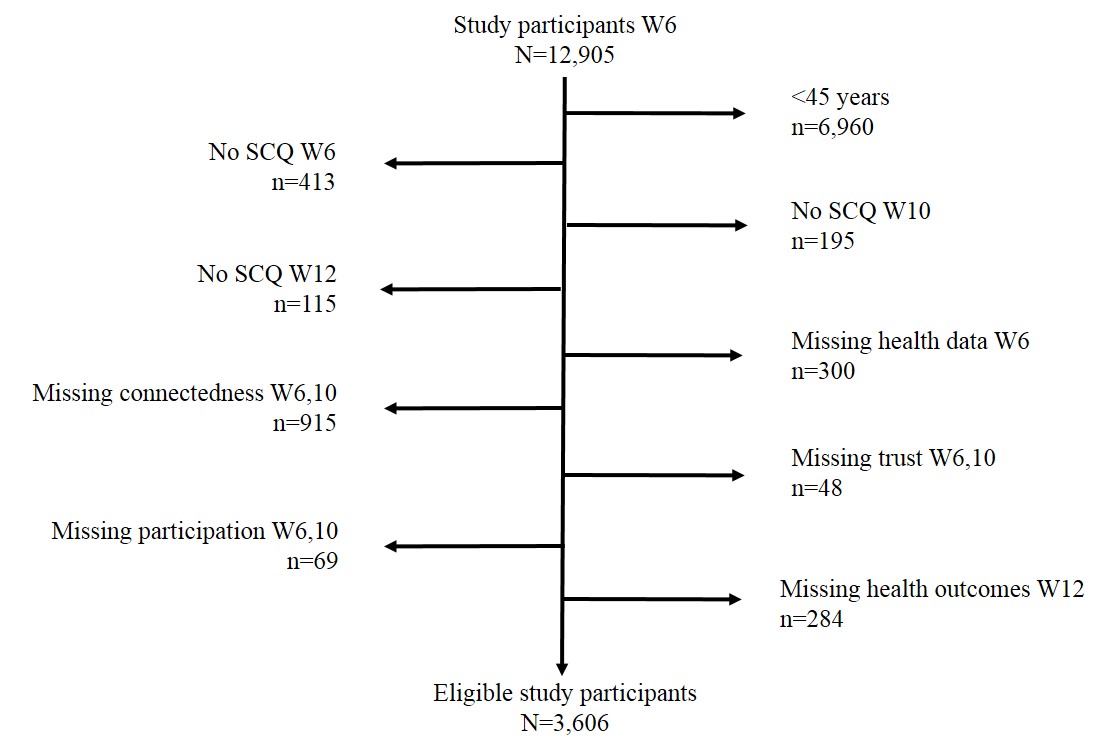


Figure S1. Participation flow diagram

**Appendix 2 Exposure and outcome measures**

***Social Capital*:** We measured three components of social capital: connectedness, trust and participation. The measures used, their response formats and the cut points used in this study are shown in Table S1.

**Table S1. Summary of social capital measures.**

| **Concept and items** | **Response format** | **Cut point for ‘low’** |
| --- | --- | --- |
| ***Connectedness*** |  | Infrequent contact with friends or family, or, neighbours are willing to help each other out |
| - How often do you get together with friends or relatives not living with you | 7 point scale: “Everyday”, “several times a week”, “about once a month”, “2 or 3 times a month”, “about once a week”, “once or twice every 3 months”, “less often than once every 3 months” | Infrequent contact with friends and family: “once or twice every 3 months” or “less often than once every 3 months” |
| - People in my neighbourhood are willing to help each other out | 7 point Likert scale from 1 “strongly disagree” 7 “strongly agree” | Neighbours unwilling to help out: disagree, with a score of 1-3 on the response scale |
| ***Trust*** |  | Disagree with a score of 1-4. |
| - Generally speaking, most people can be trusted | 7 point Likert scale from 1 “strongly disagree” 7 “strongly agree” | Disagree with a score of 1-4. |
| ***Participation*** |  | No club membership and infrequently attends community events |
| - Are you an active member of a sporting, hobby or community-based club or association | Yes or No | No club membership: “No” |
| - How often do you attend community events that bring people together? | 6 point scale: 1 “never”, “rarely” ”occasionally”, “sometimes”, “often” and 6 “very often”. | Infrequently attending community events: “never” or “rarely”. |

**Health:** We assessed three forms of health: physical, mental and self-rated health. Physical and mental health were measured using subscales of the Medical Outcomes Study Short Form 36-item Health Survey (MOS-SF-36). The physical functioning subscale measures whether respondents are limited “a lot”, “a little” or “not at all” in ten everyday activities, ranging from vigorous activities such as running, to bathing and dressing”. The mental health subscale measures frequency of positive (did you feel full of life”, “have you been a happy person”) and negative (“have you been a nervous person”, have you felt down”) symptoms experienced in the previous four weeks. For both subscales, scores ranged from 0-100 with higher scores representing better health. Age relative cut points, capturing the bottom 20% of scores for each age group, were used to define poor health. The cut points are described in Table S2.

Table S2. Age relevant cut points used to define poor health.

|  | Age group | | | |
| --- | --- | --- | --- | --- |
|  | 45-54 | 55-64 | 65-74 | 75+ |
| Health subscale |  |  |  |  |
| Physical functioning: | 0-78 | 0-62.5 | 0-50 | 0-28 |
| Mental health | 0-60 | 0-67 | 0-70 | 0-68 |

Note: Cut points represent the bottom 20% of score within each age group.
